# Use and importance of different information sources among patients with rare diseases and their relatives over time: a qualitative study

**DOI:** 10.1186/s12889-020-08926-9

**Published:** 2020-06-05

**Authors:** Svenja Litzkendorf, Martin Frank, Ana Babac, Daniel Rosenfeldt, Franziska Schauer, Tobias Hartz, J.-Matthias Graf von der Schulenburg

**Affiliations:** 1 Center for Health Economics Research Hannover (CHERH), Leibniz University Hannover, Otto-Brenner-Straße 7, 30159 Hannover, Germany; 2 Faculty of Public Health Services, Ostfalia University of Applied Science, Rothenfelder Straße 10, 38440 Wolfsburg, Germany; 3 Department of Dermatology, Medical Center, University of Freiburg, Faculty of Medicine, University of Freiburg, Hauptstraße 7, 79104 Freiburg, Germany; 4 Clinical Cancer Registry of Lower Saxony, Sutelstraße 2, 30659 Hannover, Germany

**Keywords:** Rare diseases, Information sources, Informants, Health information seeking, Qualitative research, Content analysis, Self-help, Online information, Written information

## Abstract

**Background:**

Finding reliable information on one of more than 7000 rare diseases is a major challenge for those affected. Since rare diseases are defined only by the prevalence criterion, a multitude of heterogeneous diseases are included. Common to all, however, are difficulties regarding information access. Even though various quantitative studies have analyzed the use of different information sources for specific rare diseases, little is known about the use of information sources for different rare diseases, how users rate these information sources based on their experiences, and how the use and importance of these information sources change over time.

**Methods:**

Fifty-five patients with a variety of rare diseases and 13 close relatives participated in qualitative interviews. For these interviews, a semi-structured guideline was developed, piloted, and revised. Data analysis involved a qualitative content analysis developed by Philipp Mayring.

**Results:**

The participants considered internet as the most important and widespread information source, especially for early information. Although patients have difficulty dealing with information obtained online, they consider online searching a quick and practical option to gather information. During the course of the disease, personal contact partners, especially self-help associations and specialized doctors, become more important. This is also because information provided online is sometimes insufficiently detailed to answer their information needs, which can be complemented by information from doctors and self-help.

**Conclusions:**

People rarely use just one type of source, but rather refer to different sources and informants. The source used depends on the type of information sought as well as other person-related factors such as preexisting knowledge and the disease stage. To improve people’s information searching and connect them with medical specialists in rare diseases, a central information portal on rare diseases might be a suitable access point to provide free and quality assured information for patients, caregivers, and physicians. This would allow not only patients but also doctors to find quality assured information on symptoms and therapies as well as patient associations and specialized doctors.

## Background

In recent years, rare diseases have become an important issue. Although a uniform definition is still pending, rare diseases are globally characterized only by their low prevalence. In Europe, “rare diseases” is the umbrella term for diseases that affect less than or equal to 1 in 2.000 people. Although rare diseases can differ greatly in type, symptoms, and causes, affected people usually face similar challenges. These include insufficient information. On the one hand, this is because many rare diseases are so rare, that only little information exists. Beyond that, information is often widely dispersed and difficult to find in the vastness of the internet or literature, so that access is limited [1].

However, it is undisputed that information plays an important role in coping with illness [2–8]. Based on Antonovsky’s concept of sense of coherence, perceiving the world as comprehensible, manageable, and meaningful enables an individual to cope with critical life events [9]. In this context, information can make a decisive contribution in helping individuals develop their sense of coherence [10]. Understanding an illness’s causes, symptoms, and impact seems to be a precondition for dealing with the disease in everyday life and can increase people’s quality of life. Accordingly, not being provided adequate information about the disease and its implications can lead to feelings of resignation and fear among patients and their families [5–8]. Moreover, information is an important prerequisite to know where help can be found and pave the way to specialized centers and providers [3]. Both, again, impact patients’ health situations. However, the information needs of individuals with rare diseases and their relatives include not only medical knowledge regarding diagnosis, therapy, progress, and prognosis but also information on various other aspects of the disease. These include practical information for everyday life, psychological counselling and social law aspects [3, 11]. Therefore, knowing how patients as well as their family members, who can also be strongly affected by their relatives’ disease, search for information is an important issue. To shape the information gathering process as well as possible and thus meet the patients’ information needs optimally, knowledge is needed on how searching is done, what sources are used, and what relevance different sources have. Moreover, how the use and relevance of different sources change over time should be investigated.

Information searching patterns from patients suffering from chronic but not rare diseases have been extensively analyzed. Numerous studies revealed different sources of information, which are of importance to different groups of patients, but mostly cancer patients [12–18]. These range from physicians, who are often rated as one of the most favored and trusted sources [13, 17, 18], to information brochures, the internet, as well as non-medical professionals, such as pharmacists and nurses [14–16]. Other generally used sources of information include books, newsletters, and mass media sources. For patients with common diseases, family members and friends were also used to gather disease related information [14, 16]. Moreover, some factors have been identified that affect people’s search for information. Female patients were reported to inform themselves more often and to use more sources than male patients. Additionally, younger patients and those with a higher education showed more frequent information seeking behavior than older patients and people with a lower educational background [15]. Regarding phase of illness, it was found that shortly after their diagnosis people favored written information while at a later stage relatives and friends become increasingly important [16].

Because of the specific characteristics of rare diseases, such as unpredictable courses, limited available knowledge, lack of exposure in the media, etc., it can be assumed that information seeking behaviors by people with rare diseases are not completely similar to those of patients with common diseases [19]. However, still little is known about how people affected by a rare disease and their families search for information. Previous studies may be outdated, have relied on few single sources, focused on specific information needs only, and did not focus on rare diseases in general [19–24]. Teixeira et al. [22] conducted a questionnaire survey of 1019 patients with a rare blood disorder, which showed that medical specialists are of particularly great importance when it comes to sources that were widely used for information gathering. In this study, respondents who reported feeling sufficiently informed about genetic testing and its implications for their health mostly reported having received this information from medical specialists before family doctors and support groups. Even though medical specialists were also the source they most trusted, they would like to get more information from their family doctor. Furthermore, among patients who did not feel sufficiently informed, the majority answered that they would like to gain information from their family doctor. Additionally, non-medical sources, such as patient associations, websites, nurses, and printed information were of importance to the respondents. After medical specialists, patient associations were the most trusted information source. Due to the high level of knowledge possessed by patient associations, these are often called patient experts [25].

General practitioners also proved to be one source of information patients would like to receive significantly more information from, according to a study by Matti et al. [21]. They identified preferred sources of information based on responses from 30 patients with multiple sclerosis (MS) and found that there was a discrepancy between the amount of information people actually receive and the amount they would like to obtain. Moreover, eye specialists and neurologists were identified as sources they would like to receive more information from. Regarding MS patient associations and MS specialist nurses, patients reported an almost ideal amount of information that was being provided.

An older study from Lanigan and Layton [20] on 108 patients with a rare skin disorder drew similar conclusions. The results from their questionnaire survey also illustrate that medical specialists were the most used and preferred information source, followed by general practitioners. However, it must be considered that this study occurred before the arrival of the internet, so that its relevance for today’s conditions is limited. Wibberly et al. [23] studied 16 patients with a rare lung disorder and identified various information sources by means of face-to-face interviews. These include primary healthcare physicians, patient information leaflets, as well as the World Wide Web, nurse specialists, and patient support groups. The most valuable information sources were medical specialists in rare lung diseases, nurse specialists, as well as patient support groups.

Carpenter et al. [19] also confirmed that physicians and the internet were the most used and credible sources for patients with vasculitis to obtain information on medication, followed by pharmacists, and other affected people. Based on an online survey of 232 patients, they also found that family and friends are not relevant sources of information, presumably because they do not hold much information on rare diseases. Additionally, gender differences were found. While male patients, unlike female patients, rated their spouses or partners, as well as nurses as fairly credible sources, female patients preferred medication package inserts and the internet as sources of information.

Molster et al. [24] conducted an online survey of 810 patients with different rare diseases and found that the most sought and preferred sources of information were medical specialists and patient organizations followed by friends and family members. Regarding non-personal sources, respondents stated that they prefer to be referred to an information website or social media. Other preferred types of information sources included printed media, such as leaflets and brochures, as well as journal articles.

To summarize, family doctors and medical specialists, the internet, and support groups are of great importance for patients with rare diseases when searching for information on their disease. However, limited studies have investigated the use and perceived credibility of information sources over time and if so, their reasons for it. Since existing studies are based mostly on quantitative methods, further qualitative research is needed to analyze how people with rare diseases assess different sources and on what experiences. Due to its open approach, qualitative research can achieve a deeper understanding of peoples’ attitudes and causes. The aim of this study, therefore, was to obtain a holistic picture of the information sources used by patients with various rare diseases and their relatives; specifically, what relevance they attach to different sources and how this relevance changes during the course of the disease.

## Methods

Due to the lack of substantial data on information sources in the field of rare diseases, the authors decided on a qualitative study design. Thus, it is possible to explore under-researched areas with maximum openness and reveal all aspects of importance for patients and their families concerning finding information. To detect patients’ experiences regarding information acquisition and information sources used, semi-structured interviews were conducted. Therefore, we developed an interview guideline, stimulating people to tell us about their medical history and the way they searched for information (Table 1). Since the research team included young associates with mostly theoretical knowledge in qualitative research, this was done in close cooperation with an external specialist at Hanover Medical School, who has long-time expertise in qualitative health research. The specialist conducted a workshop during which they shared the knowledge and skills required for planning, conducting, and analyzing qualitative interviews. In addition, extensive literature studies were carried out. Afterwards, the authors developed a first draft of the interview guideline and discussed it jointly with the specialist. A concerted version was then presented at a research workshop held at Hanover Medical School with several internal and external qualitative researchers, during which revisions were made to generate the final version. Individual sources of information and their usefulness could be derived from this. After pretesting the interview guideline with three patients with rare diseases, we observed that patients diagnosed before or shortly after birth found it difficult to answer the opening questions and narrate their diagnostic paths. An alternative conversation starter was then added, to ensure that it was suitable for such patients too.

**Table 1:** Semi-structured interview guide

Set	Principal questions
Experiences with the disease (from patients who consciously experienced their diagnosis)	Please remember the beginning of your disease. What changes did you notice?
	How did diagnosis proceed?
	What happened after diagnosis?
	When imagining yourself in that position again, how did you feel?
Experiences with the disease (from patients who did not consciously experience their diagnosis)	Please tell me about your disease and how life has changed due to it.
	How does your disease affect your everyday life?
	Some people want to learn more about the diseases that they live with. How about you?
Information seeking and information needs	How was that, striving to find information about your disease?
	Do you remember any events that you associate with increased demand for information?
	Please tell me about situations in which it was easy to gather information.
	Please tell me about situations in which it was difficult to gather information.
	Which moments do you consider important in searching for information?
Type of access	Please imagine the many possibilities of modern and classic media to communicate. Please recall your own situation. Which media did you use when searching for information?
	Which medium would you prefer for accessing information?
Completion	Are there any other topics that you would like to talk about?

To select a heterogeneous and balanced sample, several medical experts from the project consortium divided the total of rare diseases into eleven different groups of diseases in accordance with the affected organ systems. It was planned to conduct six interviews in each group as well as ten interviews with patients, who had to wait for at least 10 years until they received a correct diagnosis. Thus, a total sample of 76 patients was planned to be recruited. Nevertheless, upon saturation of interview data, a smaller sample would suffice. Participants were recruited consecutively over several months by a physician and GCP trained study investigator Freiburg Center for Rare Diseases (FZSE) at the University Medical Center Freiburg, University of Freiburg, Germany. Patients with rare skin diseases and their relatives were reached out directly through FZSE via personal approach during patient visits (gatekeeper sampling) and board notices (sampling by self-activation). To recruit other groups of rare diseases, more centers for rare diseases belonging to the consortium of rare diseases (AG-ZSE) were included as well as patient organizations.

Care was taken to establish a consolidated interview atmosphere with the participants. Therefore, researches allocated enough time and visited patients and close relatives at home whenever requested. Telephonic interviews were conducted only if participants requested for it. After making small talk, we explained our research project in detail and answered any questions. We emphasized that all data would be kept strictly confidential and that anonymity would be ensured, so that retroactive conclusions concerning the participants would not be possible.

The interviews were analyzed following the structured content analysis method by Philipp Mayring [26]. Each audio recording was verbally transcribed and read into MAXQDA analysis software. Subsequently, two researchers worked through the first three interviews and marked all relevant text passages. To develop an extensive system of categories (Table 2), a deductive-inductive procedure was used. Several categories could be derived from the theoretical framework based on previous research on information sources for rare diseases, including medical specialists, patient organizations, and primary care doctors. These were completed by inductive categories if they appeared from the text. This procedure was followed by a critical examination and, if necessary, modification of the original categories. Afterwards, the marked text excerpts were analyzed with regard to the research question. After assessing the interview transcripts, the researchers conducted three focus groups with participants of the interviews and one focus group with patient representatives and members of the Alliance of Chronic Rare Diseases in order to discuss and validate the findings.

**Table 2:** Coding tree

Core categories	Sub categories
Print media	Books
	Brochures/leaflets
Television	
Helpline	
Internet	Non-specified
	Patient organization
	Medical facility
	Encyclopedia
	Social media
	Scientific database
	Email/newsletter
Personal contacts	Center for rare disease/specialized clinic
	Primary care doctor
	Pediatrician
	Specialized doctor in outpatient practice
	Parents
	Social worker
	Patient organization
	Other affected persons
	Congress

Extracted citations were translated by an external translation service, approved by a native speaker and then included in the paper. The following will accompany direct interview quotations: Gender (“M” for male, “F” for female), a consecutive number, age, and status as either a patient (“P”) or relative (“R”).

## Results

We interviewed a total of 55 patients and 13 close relatives between March and December 2014 (Table 3). There were almost twice as many women (*N* = 45) as men (*N* = 23). Participants’ mean age was 50.5 years. The interviews lasted 10–143 min, with 68 min on average.

**Table 3:** Participant characteristics

Patients		
Age	Gender	Group of rare disease
23	female	Genetic skin disease
32	male	Cystic fibrosis and pulmonary disease
32	male	Immunodeficiency
39	male	Skeletal dysplasia
66	male	Genetic skin disease
85	female	Connective tissue disease
70	male	Connective tissue disease
72	male	Genetic kidney disease
47	male	Congenital metabolic disease
50	female	Immunodeficiency
53	female	Genetic skin disease
58	female	Genetic disease of the digestive tract
54	female	Cystic fibrosis and pulmonary disease
57	female	Immunodeficiency
44	female	Neuromuscular disease
43	female	Cystic fibrosis and pulmonary disease
47	female	Neuromuscular disease
71	male	Neuromuscular disease
44	female	Genetic skin disease
53	female	Connective tissue disease
72	male	Genetic skin disease
48	female	Immunodeficiency
54	female	Genetic skin disease
58	female	Congenital metabolic disease
72	female	Immunodeficiency
48	female	Genetic kidney disease
47	female	Congenital blood formation disease
44	female	Skeletal dysplasia
27	female	Congenital blood formation disease
36	female	Genetic kidney disease
40	female	Congenital metabolic disease
61	female	Neuromuscular disease
48	male	Congenital blood formation disease
44	female	Genetic eye disease
52	female	Genetic eye disease
46	male	Cystic fibrosis and pulmonary disease
60	male	Neuromuscular disease
62	female	Neuromuscular disease
48	female	Genetic eye disease
61	female	Connective tissue disease
66	female	Congenital metabolic disease
18	female	Congenital blood formation disease
64	female	Congenital metabolic disease
37	male	Cystic fibrosis and pulmonary disease
49	female	Congenital metabolic disease
59	male	Genetic kidney disease
70	male	Connective tissue disease
45	female	Genetic kidney disease
51	female	Genetic kidney disease
62	female	Genetic eye disease
39	female	Neuromuscular disease
51	male	Immunodeficiency
40	male	Skeletal dysplasia
74	male	Cystic fibrosis and pulmonary disease
69	female	Immunodeficiency
**Relatives**		
Age	Gender	Group of rare disease
44	male	Neuromuscular disease
48	male	Skeletal dysplasia
28	female	Genetic skin disease
46	female	Genetic skin disease
60	female	Skeletal dysplasia
50	male	Neuromuscular disease
43	female	Skeletal dysplasia
46	male	Congenital metabolic disease
40	female	Genetic skin disease
49	female	Cystic fibrosis and pulmonary disease
45	female	Genetic skin disease
32	female	Genetic disease of the digestive tract
41	male	Skeletal dysplasia

Based on the evaluation of the interviews, a multitude of different information sources used by patients and their relatives for gathering information on rare diseases was revealed. The authors disclosed four main themes that were of importance in nearly all interviews. These main themes include the “internet as the first source of information” (theme 1), which describes the relevance of online searches for those affected. The second theme highlights the role of patient organizations and other patients in the information retrieval process, which allow for communication at peer level. Doctors and their perception as a source of information by persons affected is illustrated in theme 3. Lastly, theme 4 deals with written information.

### The internet as the first source of information on rare diseases

Many of those interviewed reported in detail about their struggle to receive a correct diagnosis. Often this meant a long-lasting and emotionally charged odyssey. The need for information, once a diagnosis has been made, was accordingly high. In this context, for almost all the respondents, the internet and especially search engines such as Google were one of the first sources to search for information on their own or their relatives’ disease. According to the interviewees, this allowed them quick and uncomplicated access to information. In this context, different approaches to how to proceed when searching online for information were identified. Most of the participants simply googled their disease and clicked in a more or less unstructured or unskilled manner through the provided information websites, while others advanced more systematically. In many cases, it was possible to establish a connection between people’s searching approach and their prior knowledge. Patients or family members, who work in the health sector and are familiar with medical terms, demonstrated a more targeted and satisfied approach to research online than those without medical backgrounds. Moreover, people who are familiar with online searches reported fewer difficulties.

*“It was when everything was new. We took in all the information we could.”* (M47, 59 years, P).

*“Well, the information is primarily shared over the internet.”* (F14, 57 years, P).

*“( …*) *I enter it into the internet and then find the information. It would now be the easiest and quickest way for me.”* (F17, 47 years, P).

*“Well, when I am looking for something like this, I will look at Wikipedia first, because I think it’s great and well-structured. Yes, then I do not know anymore. Then you land somewhere at large. What just/ whichever link appeals to one, but I cannot recite it now.”* (F67, 45 years, R).

Even though, the internet was perceived as providing easy and quick possibilities for information seeking, most respondents did not report satisfaction with the search results at the beginning of their research. Dissatisfaction, for example, arose when only little information was available. This was particularly the case when people were affected by very rare diseases with only a few sufferers or few research efforts. Otherwise, finding a multitude of information was also challenging for searchers. Interviewees, who told us that there was a wide range of information, often felt they are not enough of an expert to manage these amounts of data. Moreover, people suffering from diseases that proceed differently in each individual case recounted problems comprehending what information is correct for specific cases. Younger persons and people who use the internet on a regular basis reported fewer difficulties with large quantities of information than those who are unskilled in online searches and of older age. Moreover, it could be seen that people reported fewer difficulties as the disease progressed and their expertise grew.

*“I also think that it is better, I think it sucks when there are several million websites when you look up cancer or the like. I also think that if someone gets diagnosed with cancer, he immediately wants to know what impact it will have. If there are then a thousand websites, you will go completely crazy.”* (F17, 47 years, P).

Another challenge reported in connection with online searches was that of dealing with information that is perceived as frightening. Many interviewees told us that when they started searching, they found information on the internet that was shocking, for example regarding life expectancy, severe courses of disease, etc. This information was so dreadful that some of our interviewees did not continue their online research. In this regard, some patients criticized being left alone with their findings and worries and wished for greater support from their doctors. Being alone with this information, in their opinion, could incite panic or despair. The results suggest that when people start searching they do not have enough expertise or support by others to put information into its proper context and assess it correctly. Our interviewees, in this connection, expressed the need for a closer support, especially by their doctors.

*“Well, I was only on Wikipedia. What I read there shocked me, because it sounded extremely bad. After that, I never went onto the internet again.”* (M60, 46 years, R).

*“You stand there alone, and that is, that is the problem, when you stand alone with your illness. Err. Meanwhile you think about it and say: Mhh. And now?”* (M38, 60 years, P).

Furthermore, peoples’ perceptions of the utility and credibility of the information found online varied greatly. This became obvious in regard to who is behind the information (website), what information is communicated, and how. Since most patients and their relatives barely know about their own or their relatives’ disease shortly after diagnosis, the assessment is based partially on who is the websites’ operator rather than on the contents of the information itself. Many of our interviewees first encountered Wikipedia when they started searching online. Some of them rejected this website, since the information offered there was too generic for them. Others criticized Wikipedia because it does not control its information, which can be changed arbitrarily by anyone at any time. In contrast, other patients and family members expressed positive views about it. From their point of view, especially in the beginning, Wikipedia is a good source of information to get an idea of the disease, its causes, symptoms, and progression. It was also highlighted that this information, compared with others, was clearly structured as well as quickly and freely available. Looking back, some people who now have an extensive knowledge on their disease rated the quality of the information offered there as good or high.

*“( …*) *and then, after the appearance, one decides what is serious, yes, who is behind it, ( …*) *are the err, here mmhhh Alliance of the chronic/well, the ACHSE associated, NAMSE associated, yes.”* (F35, 44 years, P).

*“Yes, I had, of course, I have. I then do not want useless information, because of my job I also have reasonable/ well, I would never at Wikipedia, we already had it.”* (F19, 44 years, P).

*“I just entered it and then usually ended up at Wiki. Wikipedia. It was the most reliable for me.”* (F14, 57 years, P).

Medical databases on the internet, such as PubMed, were hardly used. Often only interviewees with medical backgrounds reported knowing these sources of information. This was described as an advantage in relation to other patients who do not have medical backgrounds, due to its high quality and current information.

*“I therefore rather checked at PubMed or so, but it was of my advantage, because I have been active in the field myself.”* (M65, 40 years, R).

### Patient organizations and other affected persons – information sharing at peer level

In many cases searching the internet for information helped patients or their family members to contact patient organizations at an early stage. No interviewee reported being informed by their doctor about this way to receive support and information. Almost all our respondents who used a patient association website valued their supply of information highly. One person, however, criticized that their information was not comprehensive and current enough regarding new developments and findings. Another patient, who visited a website that was not specialized on one disease but a group of diseases also reported lower satisfaction, since there was detailed information only on the more common rare diseases. Other interviewees praised their relevant and helpful information. In particular, concerning information on issues in everyday life, such as finding medical specialists near to home, dealing with the disease in family and working environments, etc., self-help organization websites provided crucial hints. One person especially emphasized that his patient association helps to make the latest findings accessible to the general public by translating English scientific articles into German and displaying them on the website. Thus, patient organization websites contribute to knowledge transfer and access. For many of our respondents, patient association websites provided the most reliable and high-quality information, so that after identification, no further websites were used.

*“Well the main information, the thing that helped with our progress the most, was the support group. The exchange actually starts there, when you join in on the conversation at eye level ( …*). *”* (M58, 48 years, R).

*“It strengthens one, when you sometimes think you are insane. (LAUGHS) Yes, because everything changes and one thinks, yes why am I feeling so bad, why am I always tired and hurting? But when you have the opportunity to exchange stories err, then you can put your mind at rest, because you learn that, ok, it is normal.”* (F31, 36 years, P).

*“No. I never looked it up, because I have to say, up to three years ago we regularly participated at the annual meeting of the support group or the regional meeting in LOCATION and therefore the information actually was sufficient.”* (F51, 62 years, P).

Interviewees particularly valued the close personal contacts made with those committed to self-help. When a rare disease leads to similar and severe progressions and is accompanied by comparable restrictions and challenges as those of affected individuals, patient organizations play a key role in information gathering. While there is sometimes too little time for patients in the medical setting, in the self-help field patients with rare diseases and their relatives often feel that people take a lot time for their issues and needs. It was often reported that the personal contact resulted in a close and strong contact between existing members of the patient organization and the interviewees for years. Furthermore, people see information from patient organizations as an opportunity to gain practical knowledge that goes beyond the perfunctory information they receive from the internet. Since rare diseases often show an individual progression, online information is perceived as too generic, while self-help contacts meet the demands for more specific information.

*“I then called the chairman myself and he immediately took an hour of his time and answered everything, the questions, that I already had and more ( …*). *”* (M64, 46 years, R).

*“And those are the information, which the doctor does not give you, how I deal with everyday life, when I need what.”* (F22, 72 years, P).

*I: “How do you judge the quality of the information?”*

*P: “That however is good, well only the information about the support group, nothing else.”*

*I: “And the information, that you found on other sites in the internet?”*

*P: “No. It was too general, unmeaningly.”* (F10, 50 years, P).

Nevertheless, some patients feel no need for personal exchange or even reject the principle of self-help. This is based mainly on the assumption that it only serves the purpose of commiserating with each other. This can be noticed, in particular, among people who have trusting relationships with persons outside patient organizations, such as medical specialists in hospitals, who are available to answer any questions. However, individuals who are reserved about the idea of self-help due to this assumption often have no practical experience with self-help at all. Others see no additional benefits since disease progression differs too greatly from one person to the next. Moreover, people with a mild disease course sometimes do not make contact with patient organizations, since their need for information and exchange is low. They reported being able to cope with their situation and pointed out that they get along. Furthermore, meeting with patients with serious disease progression is perceived as discouraging.

*“Whining does not help; therefore, I do not sit down and moan. I do, however, understand the people that complain in the support group. Yes, I do not know if it helps them.”* (F39, 62 years, P).

### Physicians, basic health care provider and highly specialized experts

During their medical care process, patients and their families often met many different physicians. Although some patients reported receiving a quick diagnosis and were referred to specialized care from the very beginning, such as patients with cystic fibrosis, which can be easily diagnosed shortly after birth, many respondents first consulted their family doctor when searching for a diagnosis and did not attend a medical specialist until a later stage. Even in the further course of treatment, not only medical specialists, but also primary care doctors play an important role due to community care provision. The experiences with doctors outlined by the patients and their families are, however, very heterogeneous.

*“I was lucky to be under the care of a very experienced orthopedist from an early stage on ( …*). *”* (M04, 39 years, P).

*“Yes, I was not amused about it, but also not depressed. Every time I was told that it was not it, we somehow made new attempts to get a diagnosis. I have also been to a lot of so-called experts on muscles.”* (M18, 71 years, P).

### Preference for commitment and support instead of knowledge transfer from general practitioners

Many of the participants, who first contacted their general practitioner (GP), feel dissatisfied regarding information provided by their doctor. Many of our interviewees criticized that their doctor gave too little or even no information on their disease. Especially when patients received their diagnosis they complained about too little and barely patient friendly information. Even though patients and their relatives understand that doctors, who do not deal with rare diseases on a regular basis, cannot hold information about all rare diseases, they would wish for more transparent dealing with that lack of knowledge.

*“I have to say that, when it comes all doctors, ( …*) *you cannot expect anything else from them, they did not identify it, do not know this disease, that is to say, if you go there, here, my hemogram is not in order, standard things get asked ( …*). *A good doctor can recognize that a level is out of the norm, but that was of course also a little stupid, sort of, that he did not think to look into the other direction too.”* (M34, 48 years, P).

*“Yes, and there I was the one time, err, with my telephone and thought, yes, maybe the doctor will say something about it, but no, it was done for her! She had the diagnosis and it was over. I am supposed to look for someone, who mhm, yes look for a doctor.”* (F28, 47 years, P).

Patients expressed frustration and resignation with general practitioners who refused to seek assistance for their limited knowledge. Particularly, shortly after receiving a diagnosis, when specialized centers for rare diseases or contact partners had not yet been found patients felt left alone and helpless.

Nevertheless, other patients reported high satisfaction with information transmission from family doctors. In many cases, this contentedness resulted less from an immediate and comprehensive offer of information on the GPs’ part, but more from the commitment to learn more about their patients’ conditions and go in search themselves. However, even if the GP did not acquire the knowledge by himself but through the patients or their relative, this was highly valued. From the interviews, it was found that in such cases GPs often became trusted informants, near to their homes, who played an important role in patient’s health care provision.

### Specialists and centers for rare diseases – trusted and current disease-related information

For almost all interviewees involved in specialized care, such as at centers for rare diseases or university hospitals, the doctors working in these institutions are an important information source regarding medical issues. Besides patient organizations, medical specialists in these centers were often described as key informants on disease specific information. After diagnosis, as well as in the course of the disease when the state of health deteriorates noticeably or treatment becomes necessary, the need for information sharing with specialized doctors arises. Many of our interview partners reported very high information quality and valued the fact that specialized carers are available for all kind of questions. The currency of the information was furthermore praised. Because of their proximity to research efforts and other experts, medical specialists have up-to-date knowledge that they pass on to their patients, which is highly respected. One interviewee, however, complained that he would have to claim medical specialists’ information instead of doctors transmitting their knowledge by themselves.

*“For me, it is enough to have the feedback from the very knowledgeable skin clinic.”* (F01, 23 years, P).

*“Professor PERSON always tried to share his knowledge and his research with his patients.”* (F40, 48 years, P).

Of particular importance is also the fact that patients and their families normally have fixed individual contacts in the centers for rare diseases, who are entirely familiar with their disease history and symptoms. In this context, people also positively highlighted not needing to repeatedly explain their condition, which was felt as a relief. Some people also discussed longstanding and trusting physician-patient relationships arising from that, allowing for low-threshold contact, as well as quick and personal answers to all medical concerns. From the interviews, it became clear that patients and their families also see medical specialists and centers for rare diseases as a (good) complement to the range of information offered from their patient’s association. While those hold relevant and most trusted information on most issues beside medical issues, medical specialists are especially important regarding detailed aspects concerning therapy, diagnosis, etc.

### Printed information – high quality, but not up to date information and sparsely used

Even though many of the patients and their relatives in our interview sample received information to a large extent from the internet or personal contacts, others, however, reported the wish for printed information. On the one hand, this is because people appreciated the possibility of holding something in their hands, where they can look things up again, when they feel like it. This was especially emphasized at earlier stages of disease progression.

*“( …*) *I would rather need it in writing, to refer back to again.”* (F31, 36 years, P).

Shortly after diagnosis, for example, information brochures are perceived as helpful sources, since they provide comprehensive and often comprehensible information. Moreover, people reported that brochures are well suited for bringing them on the day of doctors’ appointments to give them a review of their disease. At later stages, however, brochures do not cover people’s needs for more specific and detailed information.

*“Well I also ( …*) *got the booklet, how do I deal with it myself and where can I get help from. Very good information, yes.”* (F43, 18 years, P).

*“Here you go. My husband has brought me informative literature, because I knew that he (doctor) did not know it. I pushed it into his hands and told him to read about it.”* (F31, 36 years, P).

Some of the interviewees found it helpful to read magazines offered by patients’ associations. Additionally, for those who did not actively participate in regional meetings or did not look for personal exchange, this type of information provision was important. In this connection, patients especially highlighted experience reports from other affected patients and families as valuable information.

*“The most important source of information was simply/ the newspaper of Glandula. Publicly displaying the personal experience reports that people wrote there, the stories of what they have been through, when they got diagnosed. That is what I realized and what I took in.”* (M47, 59 years, P).

Additionally, specialist books were used for information gathering, but some of the interviewees put them aside, discouraged by the medical terminology. Especially in the time shortly after diagnosis, they exceeded the capabilities of patients and relatives. People also complained that books would often not be up to date, a fact that can be important when considering that specialist books often refer to medical issues such as therapeutic options, which could be subject to frequent amendment.

*“( …*) *and that is anyway the medical terminology and how can you as a layman go and change it for yourself, or read it, it will not do, it does not work.”* (F17, 47 years, P).

*“As mentioned before, books, they definitely are not; they definitely do not have the latest insights.”* (M55, 74 years, P).

## Discussion

### Different sources for different needs

Patients affected by a rare disease as well as their relatives use a variety of different sources to keep themselves informed. In accordance with previous quantitative studies of specific rare diseases, among others, especially the internet, patient associations as well as specialist doctors play an important role when gathering information [6, 20–23]. However, to date, the types of information sources used by patients with various rare diseases and their families, how they assess these information sources, and how their value changes over time have remained unclear.

From our interviews, it was shown that initially after diagnosis, when the need for information is very high, only few patients obtained detailed and profound information from their doctors. This is in line with a study by Molster et al. [24] who reported that almost three-quarters of the surveyed patients with a rare disease received little to no information at the time of diagnosis. A systematic review investigating experiences of patients with rare diseases found that more than half of the included studies reported lack of knowledge among health professionals about patients’ rare diagnosis [8]. Most patients and relatives therefore searched online for further information and were confronted with a flood of information. To assess the quality and relevance of such information and deal with frightening information is a difficult task for laypersons. Therefore, this first step of information search is often a frustrating and intimidating experience. Contact with other affected persons can help patients and relatives to find their way through the thicket of information by placing them into a proper context and thus, gain a deeper understanding of the disease. Additionally, doctors can contribute to successful information acquisition if they face the challenges that people with rare diseases bring to their care provision openly. This includes that doctors show willingness to become acquainted with their patients’ diseases and do not leave them alone with information acquisition, especially in the initial time after diagnosis. This is underpinned by various studies [5, 11, 27–29]. Lack of involvement is common among health professionals when they lack experience in their patients’ diagnosis [27]. Particularly, when medical professionals withdraw in such cases, it can lead to feelings of resignation and insecurity [5]. Efforts to mitigate their lack of knowledge, on the other hand, are highly valued by patients with rare diseases [11].

We were also able to show that the use of different sources is not stable, but can change over time. While, for example, people regarded the internet as an uncertain source of information due to information overload in the beginning, at a later stage their perception changed as they learned a more targeted approach to search and carefully choose which websites to use. Thus, our results indicate that the importance of different sources varies depending on, among other things, the state of disease progression and the state of knowledge.

### Great potential for patient associations

The interviews have shown that patient organizations play a major role in people’s information acquisition. Previous studies of different rare diseases have confirmed the importance of patient organizations and knowledge sharing with other people experiencing the same condition [11, 19, 21–25]. As a contact partner at peer level, they can help people to cope with their disease by offering comprehensive and comprehensible information as well as guide their way to specialized care by helping patients and families to find competent caregivers from the very beginning. This way, time-wasting detours in information searches can be avoided. As described by the interviewees, patient associations can close the gap of information offered by medical specialists in rare diseases, by not only providing medical information, but information relevant to everyday life. Huyard [11] reported a similar finding among patients with one of the six rare diseases and their parents. They sought answers to questions regarding living with the disease in daily life, such as how to lead a happy life, from other affected persons [11]. Therefore, information from patient organizations should be regarded as an important supplement for information offered by patients’ caregivers. However, despite very positive self-help growth, its potentials do not seem to have been completely realized. Nowadays, there are approximately 60,000 self-help organizations with a health-related focus in Germany, but only a small number of those deal with rare diseases [30].

Although possibilities for participation have increased over the past decade, in the future self-help associations should be even more integrated to improve patients’ health care. As we have shown, no patient or relative from our interview study was made aware of the possibility of contacting a patient organization by their doctor. Under the term of “self-help friendliness” different attempts to institutionalize relationships between carers from the in- and outpatient sector and self-help associations in Germany have been made [31]. In this context, a set of commitments has been agreed to sustainably integrate self-help on a collective level into health services [32]. In the stationary sector, for example, different quality criteria have been defined to ensure a close connection between hospitals and self-help. So far, however, few care facilities have joined these voluntary collaborations. In the future, carers in the field of rare diseases should also endeavor to collaborate with patient associations. Besides opening their medical care provision to knowledge and experiences from patient organizations, they could also strengthen contacts between their patients and self-help groups and thereby support their patients’ coping processes.

However, to permanently secure patient organizations’ work, sufficient funding is required. Even though, the funds approved by statutory health insurance recently increased due to the Prevention Act (PrävG) adopted in 2015 [33], it is still unknown whether patient organizations have sufficient financial resources to sustain their important work. Especially, for self-help in the field of rare diseases, which is often characterized by local groups with a limited number of members and low public visibility, sustainable funding to maintain their services seems to be endangered; hence, further research is needed. Moreover, still little is known about the economic potential of self-help groups. The study shows that patient organizations play a major role for patients to find highly specialized care units. This is also interesting from an economic point of view. Patient organizations do not only provide information very efficiently at low cost but also provide a communication platform for patients to exchange their worries, fears, experiences, and observations. Until now, the role of patient organizations has not been assessed from a health economic point of view and, therefore, should be studied in the future.

### Online sources for quick and easy information gathering and recommendation for a central information portal on rare diseases

Besides the great potential for self-help associations, it has been demonstrated that online information is currently of crucial importance for patients and their families to gather information. It especially enables newly-diagnosed patients to search for information quickly and easily. Additionally, in more advanced stages of the disease, people rely on online information in case they need information on current developments. Dissatisfaction, however, arose due to the unfiltered flood as well as the unknown quality of the information.

Therefore, new approaches for optimizing and developing user oriented information systems are preferable. For this reason, efforts have been made to establish and implement an information portal on rare diseases (ZIPSE) [34], where patients, their relatives, as well as medical professionals can access clearly presented and high-quality information from a central web based point. Since information provides the basis for coping with the disease and receiving specialized care [2–8], such a portal can help to improve patients’ health situation sustainably. Besides increasing their quality of life, reduced doctor-hopping and targeted therapy can help to use limited financial resources more adequately. This also allows doctors, who cannot hold information on all 7000 rare diseases, to obtain information, for example, on treatment options, medication, or specialized medical colleagues when necessary. This could also help on the caregivers’ part to make their patients’ healthcare more efficient and compensate for the uneven level of information, which was often criticized in the interviews. Physicians should be conscious of their important role in people’s health care and endeavor to better inform themselves on their patients’ diseases, and give them specific assistance regarding which websites to use and where self-help contact partners can be found.

### Strengths and limitations

The purpose of this study was to gain insights into how people affected by rare diseases experienced their search for information, which sources of information they used, and how they assess different sources. We conducted interviews with an extensive sample of patients, with a variety of rare diseases, and their relatives, revealing a wide range of attitudes and opinions. Unfortunately, not all aspects that have been mentioned in the interviews could be reproduced in detail in this manuscript due to lack of space. Rather, the main themes were presented as comprehensively as possible. Therefore, supplementary observations regarding information sources and their potentials should be a topic for further publications.

It must be noted that our sample included individuals who had been living with the rare disease for many years and whose information needs may not be as high as those who have been recently diagnosed. Hence, recall bias cannot be completely ruled out. Nevertheless, living with symptoms, finding a correct diagnosis, and searching for information on the disease represent phases of great significance for patients and their relatives; thus, a sufficient ability to recall could be assumed.

Due to the qualitative approach of this interview study, it is not possible to generalize the findings to patients with rare diseases and their relatives as a population. It must be kept in mind that findings from a qualitative survey must be embedded in their spatial and temporal context [26]. However, that does not mean that they are not transferable to other people and situations. The creation context, however, must be considered when applying the findings to a new context.

Moreover, it was not possible to conduct theoretical sampling due to limited access to patients and their families as well as time restrictions. Sample recruitment was carried out by the Freiburg Center for Rare Diseases (FZSE) at the University Medical Center of Freiburg, University of Freiburg, Germany. As this center specializes particularly in the treatment of people with rare skin disorders, it was difficult to gain access to patients with other rare diseases. Nevertheless, by covering most of the planned six interviews in each group and reaching a saturation point at a later stage of the interview process, a heterogeneous and balanced sample can be assumed.

It should be mentioned that the interviews were obtained from a study of the conceptualization and implementation of a central information portal on rare diseases. This study identified the information needs of people living with rare diseases, their families, and of health professionals to integrate them into the information portal. Nevertheless, the researchers evaluated the interviews regarding important information sources in an unbiased way and with maximum openness.

## Conclusions

In our study, various information sources, such as the internet, self-help organizations, and doctors, have been confirmed as important access channels for people living with a rare disease and their families. Due to the qualitative approach, reliable statements on the reasons why, and how important they are to patients and their families have been made for the first time. Moreover, it was possible to show how the importance of different sources changes over time.

For physicians, especially those who do not deal with rare diseases daily, this does not mean they must hold information on all 7000 rare diseases, but they do need to know where to get quality assured information when necessary. For them as well as patients and their families, a central information portal, such as ZIPSE, might be an option. Interested people can find here bundled high quality information on a large number of rare diseases, which makes searching for information easier. It can also raise awareness of services from patient organizations that are of particular importance for patients and their families as they help to bring them together with specialized partners and address their need for practical everyday information as well as share experiences.

## Data Availability

The datasets generated and analysed during the current study are not publicly available due to data privacy but are available from the corresponding author on reasonable request.

## Abbreviations

AG-ZSE: Consortium of rare diseases
F: Female
FZSE: Freiburg Center for Rare Diseases
GCP: Good Clinical Practice
GP: General practitioner
M: Male
P: Patient
PrävG: Prevention act
R: Relative
ZIPSE: Central Information Portal on Rare Diseases
